# Cooperativity boosts affinity and specificity of proteins with multiple RNA-binding domains

**DOI:** 10.1093/nargab/lqad057

**Published:** 2023-06-09

**Authors:** Simon H Stitzinger, Salma Sohrabi-Jahromi, Johannes Söding

**Affiliations:** Quantitative and Computational Biology, Max Planck Institute for Multidisciplinary Sciences, Am Fassberg 11, 37077 Göttingen, Germany; Quantitative and Computational Biology, Max Planck Institute for Multidisciplinary Sciences, Am Fassberg 11, 37077 Göttingen, Germany; Quantitative and Computational Biology, Max Planck Institute for Multidisciplinary Sciences, Am Fassberg 11, 37077 Göttingen, Germany; Campus-Institut Data Science (CIDAS), Goldschmidtstrasse 1, 37077 Göttingen, Germany

## Abstract

Numerous cellular processes rely on the binding of proteins with high affinity to specific sets of RNAs. Yet most RNA-binding domains display low specificity and affinity in comparison to DNA-binding domains. The best binding motif is typically only enriched by less than a factor 10 in high-throughput RNA SELEX or RNA bind-n-seq measurements. Here, we provide insight into how cooperative binding of multiple domains in RNA-binding proteins (RBPs) can boost their effective affinity and specificity orders of magnitude higher than their individual domains. We present a thermodynamic model to calculate the effective binding affinity (avidity) for idealized, sequence-specific RBPs with any number of RBDs given the affinities of their isolated domains. For seven proteins in which affinities for individual domains have been measured, the model predictions are in good agreement with measurements. The model also explains how a two-fold difference in binding site density on RNA can increase protein occupancy 10-fold. It is therefore rationalized that local clusters of binding motifs are the physiological binding targets of multi-domain RBPs.

## INTRODUCTION

RNA-binding proteins (RBPs) regulate various steps of mRNA biogenesis including RNA splicing, localization, translation, and degradation (1). To ensure that these proteins bind the correct set of RNA molecules and at the right regions, the interactions have to be highly specific. Yet many RNA-binding domains (RBDs) bind to short and degenerate RNA motifs, often three, rarely more than five nucleotides in length (2,3), and the dissociation constants (*K*_d_) of their RNA-binding domains are often in the micromolar range, sometimes hundreds of micromolar (4–11). In contrast, single DNA-binding domains typically recognize somewhat longer motifs (12–14) and the dissociation constants of most transcription factors are in the nanomolar range.

**Figure 1.**
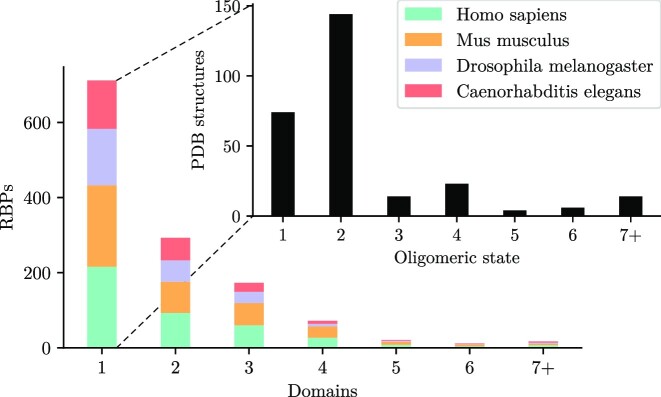
Most RBPs have more than one domain per chain or per homooligomeric complex. Numbers of RNA-binding domains per protein for proteins in the RNA-binding protein database (RBPDB) (17), which contains proteins from human, mouse, Drosophila and Caenorhabditis elegans. Inset shows in black the oligomeric state as predicted by the PDBePISA tool (18) for the 279 PDB structures of 136 of the RNA-binding proteins with only one domain.

Despite the low affinity of the individual RNA-binding domains, cooperativity between multiple domains in an RBP can result in high specificities and avidities (defined as an ‘effective’ association constant, see Materials and Methods) for the entire RBP much higher than the *K*_a_s of individual domains (15,16). When RBPs form oligomers or polymers, all RNA-binding domains of the complex can bind RNA cooperatively. Roughly 80% of eukaryotic RBPs either have at least two binding domains (17) or assemble into homooligomeric complexes with multiple RNA-binding domains (18) (Figure 1).

The increase in avidity via cooperative binding can be explained by the high local concentration of a protein binding domain at the second RNA site when the first binding domain is bound to the first RNA site, which adds to the background concentration (19,20). We will show here that, when this local effective concentration *c*_eff_ is *x*-fold higher than the *K*_d_ of the still unbound binding site (in isolation), the effective *K*_d_ for this interaction can be *x*-fold lower than for the isolated RNA-binding domain.

Thermodynamic models of cooperative binding for two binding units have been developed for binding of bivalent antibodies to antigens (19,21), of ligand binding by bivalent and multivalent receptors (22,23), and of DNA-binding proteins with two DNA-binding domains (24). In all of these cases, the combination of multiple binding domains and target sites, and their connection through flexible linkers increases avidity in an analogous way to multi-domain RNA-binding.

To better understand cooperative RNA-protein interactions and the biological implications that arise from cooperativity, we need to model quantitatively the avidity of proteins or oligomeric complexes with more than two RNA-binding domains. So far, existing models have only described cooperative binding between two domains, with flexible linkers between the domains of one binding partner (20).

Here, our goal is to develop a simplified model that can provide biologists and biochemists insight into the important effects of cooperative binding of multi-domain RBPs. Our goal is not to develop a model that can make accurate predictions of avidities as this would require, if at all possible, detailed atomic-level molecular dymamic simulations.

We present an equilibrium thermodynamic model for multi-domain RNA-binding with any number of RNA-binding domains. We treat the RNA linkers between binding motifs as worm-like chains and, in contrast to earlier work (19,20), we take the entropy of the chain into account. However, we have to simplify by ignoring interactions of the RNA linker with the proteins. The model can describe RNA-binding domains connected by flexible peptide linkers (25), which we also treat as worm-like chains.

Using this model, we can show that the avidity increases exponentially with each added pair of binding domain and target site. In this way, high affinities and specificities can be achieved with low-affinity and low-specificity RNA-binding domains. We validate the model on seven RNA-binding proteins for which the affinities of the entire protein and of individual domains have been measured. We find that the avidities estimated with the model are in good agreement with the measured values. Lastly and most importantly, we demonstrate that, by cooperative binding with multiple RNA-binding domains with the same binding preferences, RNAs can be sensitively distinguished based on their binding motif density. This result suggests that sequence-specific RBPs achieve high specificity and avidity by binding to clusters of binding sites on their target RNAs.

## MATERIALS AND METHODS

### Simple cooperative binding model

**Figure 2.**
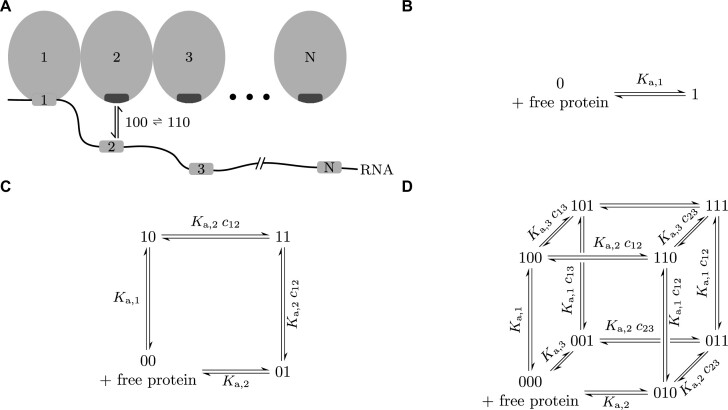
Thermodynamic model for cooperative RNA-protein interactions. (**A**) Illustration of an RNA with an RNA-binding protein binding to it. All binding sites on the RNA are only bound by one domain of the RBD. Each of these interactions has its individual *K*_a,*i*_. (**B–D**) Reaction networks for one, two, and three binding sites on the RNA. Each system has 2^*n*^ possible states. Every possible reaction step has an association constant equal to the individual *K*_a,*i*_ for the domain-to-RNA-site interaction multiplied by the concentration of the domain at its cognate site.

The model describes the cooperative, multivalent binding of RNA-binding proteins possessing *n* RNA-binding domains to an RNA with *n* binding sites (Figure 2A). To be able to analytically calculate the avidity for the protein and its RNA substrate, we need to make three simplifying assumptions. First, we assume that each RNA-binding domain can only bind to a single, cognate binding site on the RNA, so domain 1 to RNA site 1, domain 2 to RNA site 2, and so on. Second, we assume that an RNA is at most bound by a single protein. This is a good approximation as long as the local concentration of domains of the already bound protein at the RNA sites is much larger than the background protein concentration. When the linkers between binding sites on the RNA are short enough, typically up to about 20 nucleotides, the first-bound protein will outcompete all other proteins from binding to its RNA. Third, we assume that the RNA linker between motifs does not interact with the proteins nor other parts of the RNA.

We denote binding configurations in this model by a binary string that indicates which sites are bound. For instance, 101 represents the configuration in which the first and third sites on the RNA are bound by the first and third domains of one protein.

### Inter- and intramolecular reactions of first and second order

We have to consider two types of reactions. First, when the RNA and protein are not linked, all possible reactions are second order intermolecular reactions between one protein domain and its cognate RNA binding site. We call the association constant for this *K*_a,*i*_ (units of molar), where *i* is the index of the interacting domain and RNA site. These reactions only depend on the concentrations of free RNA, [0...0], and free protein, *c* (Figure 2B).

**Figure 3.**
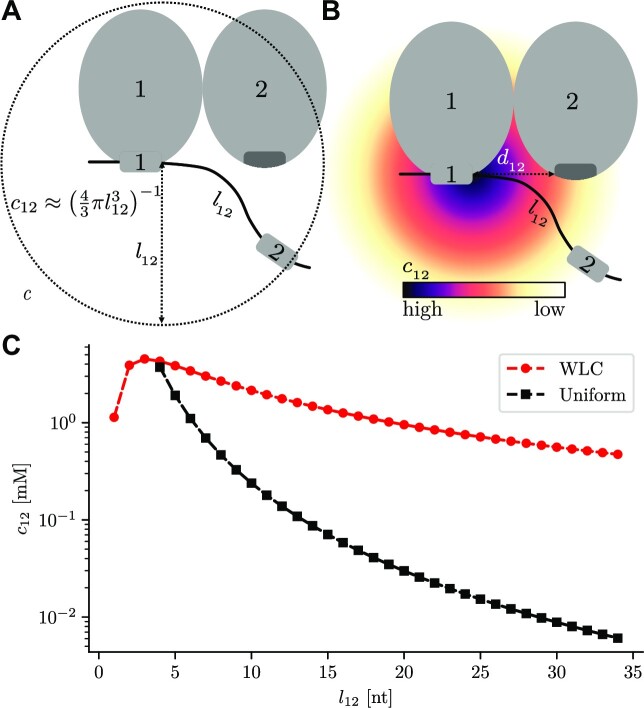
Effective concentration *c*_12_ of domain 2 at RNA site 2, when at least one RNA site is already bound. (**A**) In the simplest approximation, the concentration is uniform inside the sphere of radius *l*_12_ around the bound domain. *l*_12_ is the RNA chain length between binding sites. (**B**) More realistically, when the RNA chain is treated as a ‘worm like chain’ the concentration *c*_12_ has a Gaussian density (for large *l*_12_). Its size depends on *l*_12_ and the 3D distance *d*_12_ between binding domains on the protein. (**C**) Comparison of the effective concentration in the simple case described in (A), with the more realistic worm-like chain in (B). The concentration decays much more slowly if modelled by a Gaussian. The distance between binding sites on the protein is *d*_12_ = 3 nm.

In the second case, where the protein is already bound to the RNA with at least one domain, new domains can bind in a first order intramolecular reaction and we can describe the unitless association constant for one binding step based on the law of mass action. For example, the reaction $100 \rightleftharpoons 110$ (Figure 2A) depends on the local effective concentration *c*_12_ of domain 2 (of the already bound protein) at RNA site 2. In a first, rough approximation, we can assume this concentration to be constant inside the volume accessible to RNA site 2 (19,20). The concentration is 1 divided by the accessible volume, a sphere with radius equal to the length *l*_12_ of the RNA between sites 1 and 2 (Figure 3A): $c_{12} \approx \left(\frac{4}{3} \pi l_{12}^3 \right)^{-1}\, .$ (See below for a refinement of this estimate.) This is the same as *c*_21_, the concentration of protein site 1 at RNA site 1 when a protein site 2 is bound to RNA site 2.

The law of mass action for the reaction $100 \rightleftharpoons 110$ reads


$$\begin{eqnarray*} K_{\text{a,}2} = \frac{[110]}{[100] \: c_{12}} \end{eqnarray*}$$

and by rearranging we get for the association constant of the reaction $100 \rightleftharpoons 110$,


(1) $$\begin{equation*} K_{\text{a}, 100 \rightleftharpoons 110} = \frac{[110]}{[100]} = K_{\text{a,}2} \: c_{12}\, . \end{equation*}$$

This means that all possible first order reaction steps have an apparent association constant (e.g. $K_{\text{a}, 100 \rightleftharpoons 110}$) equal to the individual *K*_a,*i*_ for the domain-to-RNA-site interaction multiplied by the local concentration *c*_*ij*_ of the domain at its cognate site (Figure 2A).

### Thermodynamic definition of the avidity *K*_av_

We would like to calculate for each concentration *c* of the RNA-binding proteins what fraction of RNA molecules is bound by a protein. For a single binding domain and RNA binding site *n* = 1, we can simply write the association constant of binding as $K_{\text{a},1} = \frac{[1]}{c \: [0]}$, where [0] is the concentration of unbound RNA and [1] is the concentration of bound RNA (Figure 2B). When the RNA contains two binding sites and the protein contains two cognate ones (*n* = 2), we do not have a single bound state anymore but rather three: 10 (first site on RNA bound), 01 (second site bound), and 11 (both sites bound by protein) (Figure 2C). An association constant can only describe the equilibrium between two states. We therefore need a generalization of association constants to multistate systems. Following Kitov *et al.* (23), we can define the avidity—sometimes called ‘effective’, ‘apparent’, or ‘functional’ affinity or association constant—as the ratio of the sum of concentrations of all bound states divided by the concentrations of the two unbound species *A* and *B*:


(2) $$\begin{eqnarray*} K_\text{av} = \frac{[\text{all bound states }AB]}{[A]\, [B]}\, . \end{eqnarray*}$$

For instance for the case of *n* = 2 RBDs per protein and two RNA binding sites per RNA, this gives us


(3) $$\begin{eqnarray*} K_\text{av} &= \frac{[10]+[01]+[11]}{c \: [00]} = \frac{[10]}{c \: [00]} + \frac{[01]}{c \: [00]} + \frac{[11]}{c \: [00]} \, . \end{eqnarray*}$$

With the exception of Kitov *et al.* (23), the term avidity has so far mostly been used qualitatively to describe cooperativity in multivalent binding (16,26).

By substituting all concentration terms in equation (3), we can express the *K*_av_ with *n* = 2, in terms of the associations constants of the individual domain-to-RNA-site interactions *K*_a,*i*_


$$\begin{eqnarray*} K_{\text{av}} = K_{\text{a,}1} + K_{\text{a,}2} + K_{\text{a,}1} \: c_{12} K_{\text{a,}2} \, . \end{eqnarray*}$$

The derivation for this has been shown elsewhere before. In the Supplementary Methods (Section 4) we derive the *K*_av_ for *n* = 2 for the alternative case, where the two domains have the same specificities such that each of them can bind to any of the two binding motifs on the RNA.

Mainly, however, we generalize the derivation to any number *n*. Detailed mathematical steps are shown in the Supplementary Methods (Section 1), while here, we focus on explaining the intuition behind the formulas. First, we need to write equation (2) for the reaction system with 2^*n*^ states (shown in Figure 2B-D for one, two and three sites). By the same logic that leads to equation (1), we can substitute all concentration terms in equation (2). In the limiting case where the fully bound configuration dominates the partially bound state, that is, if *K*_a,*i* − 1_ *c*_*i* − 1, *i*_ ≫ 1 and *c*_*i* − 1, *i*_ *K*_a,*i*_ ≫ 1 for all *i* = 2, 3, …, *n*, we find that (Supplemental Methods, section 1)


(4) $$\begin{eqnarray*} K_{\text{av}} &\approx K_{\text{a,}1} \: c_{12} K_{\text{a,}2} \: c_{23} \: \dots \: K_{\text{a,}n-1} \: c_{n-1,n }K_{\text{a,}n} \, . \end{eqnarray*}$$

Each added binding site approximately multiplies the avidity by a factor *c*_*i* − 1, *i*_*K*_a,*i*_. Intuitively, this is a consequence of the reaction path from the unbound state [0…0] to the fully bound state [1…1], for instance by flipping unbound sites to bound sites in the order from leftmost to rightmost site. The total *K*_a_ of such an *n*-step reaction (where the total reaction is the sum of individual steps), is the product of association constants of individual reaction steps.

### Effective concentrations using the worm-like chain model

The effective concentration *c*_*ij*_ of site *j* on the RNA at site *j* of the protein when site *i* is already bound was approximated above as the reciprocal of the accessible volume $(4 \pi \, l_{ij}^3/3)^{-1}$, where *l*_*ij*_ is the chain length between binding sites *i* and *j* (20). This approximation neglects the entropy. The closer *d*_*ij*_ is to *l*_*ij*_, the fewer spatial conformations are available to the linker. For a more accurate estimate, we use the worm-like chain model, a statistical mechanics description of semi-flexible polymers (27,28). Given a sufficient length *l*_*ij*_, the local concentration *c*_*ij*_ has a Gaussian shape centered around site *i* (Figure 3B) (29). Its variance depends on *l*_*ij*_ and on the 3D distance *d*_*ij*_ between binding domains on the protein. The rigorous mathematical description of this case and of the second case in which the protein has flexible linkers between domains that is allowed to move independently is given in the Supplementary Methods (Section 2 and 3, Figure S1).

When we consider the dependence of *c*_*ij*_ on the linker length *l*_*ij*_, it is instructive to observe the difference between both models (Figure 3C). From a uniformly distributed concentration, one would expect the concentration enhancing effects of an RNA or protein linker to vanish much more quickly, compared to the worm-like chain model. According to this, cooperative binding can be observed even for RNAs with relatively long linkers between binding sites.

### Effect of different RNA motif densities

Consider a long RNA with *N* binding sites and proteins with *n* binding sites. We can estimate the avidity for proteins to bind the RNA in this special case, by making additional simplifying assumptions. First, we assume that all binding domains bind to the same binding motifs, and we model the binding sites on the RNA with equal distances between them. Second, we assume that fully bound conformations with domains bound to adjacent binding motifs dominate the *K*_av_ (Eq. (4)). The number of binding conformations for an RNA with *N* motifs is then approximately *N* − *n* + 1 higher than for an RNA with *n* motifs, because each of the conformations with all domains bound can be placed at *N* − *n* + 1 positions. Therefore, the avidity for the RNA with *N* binding sites is approximately


(5) $$\begin{equation*} K_\text{av}(N,n) \approx K_\text{av} \cdot (N-n+1)\, . \end{equation*}$$

### Simulation of cooperative binding with Gillespie algorithm

We cross-checked our analytical calculations described above with simulations using the Gillespie algorithm (30,31), implemented in the Python library Gillespy2 (32). We performed simulations of the model by defining all binding configurations as molecular entities in the simulation and determining the avidity based on trajectories of the simulated system (See Supplementary Methods, section 5 for more details on how the simulations were set up).

### Determining the model parameters

*K*_d_ values of individual binding domains are taken from experimental measurements like electrophoretic mobility shift assays (EMSA) or isothermal titration calorimetries (ITC). Distances between binding sites on the protein are 3D Euclidian distances calculated based on available PDB structures. The contour lengths of ssRNA linkers between binding sites and the length of flexible linkers between protein domains are estimated as the number of nucleotides or amino acids multiplied with a length per base of 5.5 Å (mean of 5 measurements) (33–37) or a length per amino acid of 3.8 Å (38) respectively. The persistence length *l*_p_ of ssRNA is estimated as 2.7 nm, the mean of five publications (33–37), and the mean persistence length for disordered proteins is 3.04 Å (38).

## RESULTS

### The model correctly estimates dissociation constants

**Figure 4.**
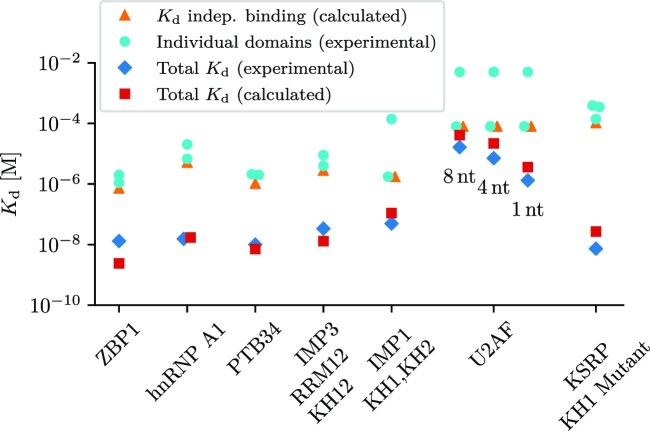
Measured avidities are in good agreement with model predictions. We found seven RBPs composed of two or three RBDs (or RBD pairs) for which dissociation constants of the full-length protein had been measured together with those of individual RBDs (4–11,39). We used the simple thermodynamic model to estimate the avidities of the full-length RBPs from those of their individual domains and from linker lengths *l* and protein binding site distances *d* and found agreement within a factor of ∼5. No free fitting parameters were used (see Supplementary Methods, section 6 for details). Orange triangles indicate the theoretical case of independent binding of the two or three domains (equivalent to an infinitely long RNA linker), calculated as the sum of *K*_a_ values of individual domains.

To validate the new model, we analyzed seven multi-domain RBPs for which the *K*_d_ values of individual domains and the whole protein have been measured experimentally. We estimated the avidity for the full-length proteins using the dissociation constants of the individual domains and employing the analytical results outlined in the Supplementary Methods, section 1 (Figure 4). We cross-checked the calculations with simulations using the Gillespie algorithm.

The proteins used are the zipcode binding protein 1 (ZBP1) (4), the heterogeneous nuclear ribonucleoprotein A1 (hnRNP A1) (5), the two terminal domains of the polypyrimidine tract binding protein (PTB) (6,7), the first four domains of the insulin-like growth factor 2 mRNA-binding protein 3 (IMP3 or IGF2BP3) (8), the first two KH2 domains of IMP1 (10), the U2 snRNP auxiliary factor (U2AF65) (11), and the K-homology splicing regulator protein (KSRP) (9,39) (see Supplementary Methods, section 6 for parameters used in the calculations). With the exception of IMP3 and KSRP, these proteins consist of two rigidly linked domains. In contrast, IMP3 consists of three domain pairs with flexible linkers between the pairs. In our model the first two of the three IMP3 domain pairs were represented as two binding sites, connected by a flexible linker. KSRP contains four KH-domains, with the middle two connected as a rigid unit. Measurements were done for the wild-type protein and for variants, in which mutations in the binding domains remove the ability to bind for that domain (see Supplementary Methods, section 7 for further assumptions we make, and predictions of the remaining measurements).

The measurements were done using fixed target RNA sequences. The affinity of full-length U2AF was measured for RNAs with three different linker lengths between the binding sites. This allows us to confirm the distance dependence in our model for the local concentration (Figure 3C). All predictions were at least within a factor ∼5 of the experimental value, demonstrating the applicability of the model to multivalent, cooperative binding of RBDs to their RNA substrates.

### Avidity increases exponentially with number of binding sites

**Figure 5.**
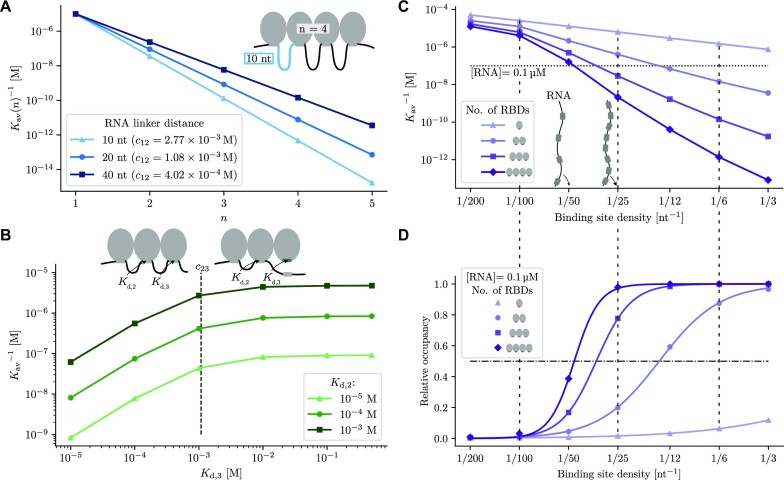
Dependence of the avidity (effective association constant) of RBPs on the number of RBDs, their *K*_d_’s, the effective local concentrations *c*_*ij*_, and the binding site density on the RNA. (**A**) The inverse avidity $K_\text{av}^{-1}$ decreases exponentially with the number of binding domains *n*, because each added binding site multiplies the avidity by ∼*K*_a, *i*_ times the local concentration *c*_*i* − 1, *i*_ of the free *i*’th RNA binding site at the site of the *i*’th free RBD (equation 4). The slope thus depends on the spacing of binding sites via *c*_*i* − 1, *i*_. All RBD *K*_d_’s were set to 10 μM and distances *d* between rigidly linked binding domains to 2 nm. (**B**) Individual RBDs contribute proportionally to the total avidity as long as their *K*_d, *i*_ is less than the local concentration, $K_{\mathrm{d},i} = K_{\mathrm{a},i}^{-1} \,<\, c_{i-1,i}$ (here shown for *i* = 3). The *K*_d_ for the first domain is *K*_d, 1_ = 10 μM, the *K*_d_s for the second and third domain are varied as indicated. $K_\text{av}^{-1}$ was calculated for equal distances between rigidly linked binding domains of 2 nm and an RNA linker length of 20 nt. (**C**) The inverse avidity decreases with the binding site density on the RNA. For this plot, we approximately neglect non-sequential binding modes, which are much less populated than the sequential ones. *K*_d_s of individual domains were 50 μM and the total RNA length was 200 nt. The horizontal line indicates a concentration of 0.1 μM used for the calculations in (D). (**D**) Binding probability of RBPs as measured by $[\text{RNA}]/(K_\text{av}^{-1}+[\text{RNA}])$ as a function of binding site density on the RNA at an RNA concentration of 0.1 μM (horizontal line in (C)). Curves show fits with sigmoidal Hill-functions, with Hill coefficients of *h*_1_ = 0.99, *h*_2_ = 2.35, *h*_3_ = 4.01 and *h*_4_ = 5.7 for one to four domains, respectively. Note the strongly cooperative, switch-like behaviour for *n* = 4 RBDs.

We then asked how the avidities for RBPs depend on the number *n* of their RBDs (Figure 5A). We chose *K*_d_ values for RBDs and linker lengths in the ranges of typical RBPs. We observed an exponential increase in avidity with the number of binding sites by a factor *K*_a,*i*_ *c*_*i* − 1, *i*_ for each added domain (eq. (4)) (i.e. a shift in the concentration at half occupancy by the inverse of this factor). The local concentration of the RBDs, *c*_*ij*_, depends on the linker length *l* between consecutive binding sites and the distance *d* between the consecutive RBDs, which determine the variance of the Gaussian concentration density (Figure 3B, Supplementary Methods, section 2). While the factor in real RBPs will depend on individual *K*_d_s and distances between binding sites, the analysis shows that the inverse avidity can drop by orders of magnitude per domain added. So, the addition or removal of one domain—or one RNA binding site—can make the difference between binding and essentially no binding.

### Contributions of individual domains to the avidity becomes negligible after a threshold in the individual *K*_d_

To further investigate the effect of domain *K*_d_s to the total affinity, we calculated the avidities for artificial RBPs with 3 domains, kept the *K*_d_ of the first domain constant and varied *K*_d, 2_ and *K*_d, 3_ (Figure 5B). As expected, the inverse avidity increases when the *K*_d_ of one individual domain is increased. According to equation (4), when *K*_a, *i*_ *c*_*i* − 1, *i*_ ≤ 1, or, equivalently, *K*_d, *i*_ ≥ *c*_*i* − 1, *i*_, the contribution of domain *i* to the avidity quickly saturates (vertical line in Figure 5B), which was also concluded from experiments in (40). Only domains with a dissociation constant below the effective concentration contribute significantly to the avidity. As Figure 3C shows, this concentration can lie in the millimolar range.

### Protein binding can depend sensitively on the density of binding motifs on the RNA

The combination of multiple RNA-binding domains is important for providing the specificity needed to bind to the correct target RNAs (41). The density of binding sites on the RNA molecule is also an important determinant of binding affinity and specificity (42,43). To investigate this effect, we calculated the avidity and the binding probability (or relative occupancy) of RBPs as a function of the binding site density on the RNA based on equation (5) (Figure 5C and D). With increasing binding site density, the RNA linker length *l* between binding sites decreases, the standard deviation of the Gaussian density of the local concentration *c*_*ij*_ decreases, and the local concentration increases. Suppose the increase is *c*_*ij*_ to $c^{\prime }_{ij}$. The avidity increases with increasing motif density by a factor $c^{\prime }_{ij}/c_{ij}$ for each of the *n* domains, or $(c^{\prime }_{ij}/c_{ij})^n$ for the whole protein, as long as the approximation in equation (5) holds, that is, as long as *K*_a, *i*_ *c*_*i* − 1, *i*_ ≫ 1. Therefore, the inverse avidity decreases approximately exponentially with increasing binding site density (Figure 5C). With growing number of domains, this results in lower threshold densities of the binding curves and more and more switch-like binding behaviour (Figure 5D). To quantify the cooperativity of this transition, we fitted a sigmoidal Hill function 1/(1 + (*D*_0_/*D*)^*h*^) to the binding probability as a function of the binding site density *D* on the RNA. The Hill coefficient *h*, a common measure of cooperativity, grows somewhat faster than the number of domains (*h*_1_ = 0.99, *h*_2_ = 2.35, *h*_3_ = 4.01 and *h*_4_ = 5.7 for one to four domains, respectively).

## DISCUSSION

### Thermodynamic model extends previous models of cooperative binding

Previous models treated cooperative binding for two binding sites. Crothers and Metzger developed a model to determine the avidity of the two binding sites of an antibody, estimating *c*_eff_ with the particle-in-a-sphere model (Figure 3A) and assuming that the RNA binding site is uniformly distributed inside a sphere with a radius of *l* around the first already bound binding site (19). This model has been extended several times, taking into account different properties like chain length of the flexible linker between binding sites/domains and also transferring it into the context of RNA-binding (20–22,24). All of these studies, derive avidities for two domains. The results for *n* = 2 match our model, which describes binding for an arbitrary number of binding sites. Previous models can only describe a flexible linker between the binding sites on one binding partner. However, many RNA-binding proteins have flexible peptide linkers between their domains. We have therefore extended the model to include the possibility of flexible linkers in both binding partners.

### Simplifying assumptions limit model accuracy

We describe a simple, idealized model system. Still, the model estimates of the avidity for the full-length proteins agree with the experimental measurements to within an order of magnitude (Figure 4). This supports the general validity of the model, but also highlights the limits in the use as a predictive tool, while it can rather offer intuitive mechanistic insights.

Various simplifying assumptions can potentially explain the deviations from measurements. Most notably, many linkers between RNA binding sites are very short. To estimate the effective local concentration *c*_eff_, we use the assumption that the chain length is much larger than the persistence length (*l*_*p*_, measure of flexibility in the worm-like chain model) of the RNA, *l* ≫ *l*_p_. If the chain length is shorter, the end-to-end distribution will not be an isotropic Gaussian anymore but will depend on the initial tangent orientation of the bound end (29). It has been shown that only for $\frac{l}{2l_\mathrm{p}} >5.6$ the distribution has a single maximum near the origin in direction of the initial orientation and approaches a Gaussian for larger values (29). The chain lengths in the examples given earlier correspond to rather stiff chains. Depending on the orientation of the next binding site in relation to the first, the effective concentration and consequently the avidity can be over- or underestimated. To increase the accuracy of estimates for *c*_eff_ we would have to take into account other geometric properties of the protein in addition to the distance between binding sites. However, for short polymers, the analytical solution to the worm-like chain model becomes highly complex and the simplicity and intuition of the model would be lost.

In addition to short RNA linkers, RNA secondary structure and unspecific binding can decrease the accuracy of the predictions. Furthermore, the sequence of the RNA influences its flexibility. Many measurements of the persistence length of ssRNA have been done with repetitive sequences. Thus, for short chains the RNA sequence might have a stronger effect on estimations of RNA flexibility, while for longer chains this effect will most likely average out.

We describe two examples in which our simplifying assumptions are violated and our model fails to accurately predict the *K*_d_ of the full-length protein. For the two-domain protein TDP-34, which binds to UG-rich RNA, our model underestimates the *K*_d_ by more than an order of magnitude because it violates two assumptions. First, since binding is measured against a (UG)_6_-RNA, it does not contain two well defined binding sites, but instead a continuous interaction surface. Second, *K*_d_s for the individual RRMS were only measured for (UG))_3_- and (UG)_6_-RNA and even vary across studies (44,45), and it is unclear whether they represent the true effective *K*_d,1_ and *K*_d,2_ in the complex. A second example is the binding of PTB (7) to different GABA RNA constructs (46). The RNAs are relatively long and the lack of defined binding sites, the complex RNA secondary structure, and the possibility for multimerization of PTB and thus, the formation of complexes with stoichiometry other than 1:1 render our model inapplicable.

### Disorder in RNA binding domains

We model two distinct situations with respect to the linkers between RBDs. In the first case, protein domains are rigidly linked and move together as a unit. In the second case, they are connected by a flexible linker and move independently, only restricted by the length of the linker. In reality, however, it is possible to observe situations in between these two extreme cases. Flexible protein linkers might either come in contact with the RNA, play a role in conformational changes of the two domains relative to each other, change their flexibility upon binding, or undergo a disorder-to-order transition (25,40). We do not expect these additional complexities to influence the general derivation of our model. Rather, all these situations require more complex calculations of the effective concentration *c*_eff_, as the assumption of either completely independent or joint movement is violated.

Partial binding of the peptide linkers to the RNA after binding of one domain violates our model’s assumption of independent movement of the RNA and unbound protein domain connected by the linker. A positive correlation could considerably increase the local concentration of the RNA binding motif at the second domain relative to our model’s estimate. In addition, the binding can result in a much reduced flexibility of the linkers. If the persistence length of RNA or peptide becomes to large, the distribution cannot be assumed isotropic, resulting in an increase or decrease of the effective local concentration of the RNA motif at the second RBD (see discussion above).

In addition to disordered linkers between domains from the same protein, intrinsically disordered regions can also lead to the association of RBDs from different proteins. This creates the possibility for cooperative binding in a similar way to what is described here. If two domains associate via their IDRs before binding to an RNA and this complex is stable on the timescale of RNA binding, the two domains can be treated in the same way as a two-domain protein, with a flexible linker between the domains. Increases in avidity are expected, whether RBDs are covalently linked or whether the effective number of domains is increased by dimerization or multimerization.

### Multi-domain RBPs can distinguish sensitively between RNAs with different binding site densities

Analyses of high-throughput measurements of RNA binding affinities for 86 RNA-binding proteins by high-throughput RNA SELEX (47), 78 by RNA Bind-n-Seq (2), and 205 by RNAcompete (3) showed generally low enrichment factors of the most enriched motifs. Enriched motifs were short and degenerate for a substantial fraction of proteins and often motifs consisted of short mono- or dinucleotide repeats (48). Our thermodynamic model of cooperative binding explains how such degenerate motifs bound with relatively low binding affinities in the micro- to millimolar range can yield highly selective binding behavior to dense clusters of binding motifs, in which density as much as binding affinity of individual motifs determines the binding affinity. This underscore the need for bioinformatic methods that can learn ‘clustered motif’ binding models for multi-domain RBPs from high-throughput experiments.

Four RBDs result in a Hill-like coefficient of 5.7 for the dependence of avidity on motif density. It is easy to imagine how homodi-, and -multimerization of RBDs can increase the effective number of RBDs to much higher numbers, particularly in liquid phases enriching for certain RBPs (next subsection). Such homo-oligomer assemblies can become exquisitely specific and affine for target RNAs with a corresponding number of target binding site.

As an example, in a study of the function of Nrd1/Nab3 heterodimers in recognizing and degrading antisense transcripts in yeast it was found that a mere factor 1.5 higher density of Nrd1 and Nab3 binding sites on antisense versus sense transcripts seems sufficient to selectively degrade antisense transcripts (43). It was later observed that, while the Nrd1/Nab3 dimer contains only two RNA-binding domains, both proteins contain disordered regions prone to form aggregates or even liquid droplet phases and that aggregation of Nrd1/Nab3 via these disordered regions leads to their polymerization or aggregation in concert with binding to their target RNA (49,50). The high effective number of binding domains in the formed polymers could therefore explain how high Hill coefficients can be realized (Figure 5D). Similarly, selective inhibition of polyadenylation of U1A mRNA over other mRNAs by U1A, depends on the presence of two binding sites on the RNA with correct spacing, to allow two interacting U1A molecules to bind (51).

Figure 5C demonstrates that four RNA-binding domains achieve an avidity of around (2 nM)^−1^ when each of the domains has a very modest single-domain RNA-binding affinity of (50 μM)^−1^. This might be the reason why RBPs rarely contain more than four RNA-binding domains: the resulting avidities would simply be below what is needed in the cell.

Some motifs on the RNA consist of mono- or dinucleotide repeats, creating the possibility for multiple binding registers in one RNA motif (15,16). This can be seen for example in the HuR C-terminal RRM binding to AU-rich RNA regions (52) and also in PTB, one of our examples, which binds to polypyrimidine tracts (53). When the repeat regions are long enough, the protein domains can bind in more than one arrangement. The effects on the affinity of an individual domain by encompassing *N* binding registers in one RNA motif can be estimated through a simple statistical consideration by dividing the *K*_d_ by a factor of *N* (equation (5) can be applied here).

The concept of ‘fuzziness’ describes the more general situation when every RNA binding site can at least to some degree bind to every protein domain (54). We calculate this effect in our model for two binding sites (Supplementary Methods, section 4). Including fuzzy binding in the calculations increases the number of possible bound configurations and thus the complexity of the combinatorics. However, it does not qualitatively change the results that we present here.

### Multi-domain RNA-binding can promote phase separation

Phase-separated biological droplets/condensates, which function to concentrate and organize molecules inside the cell, form via multivalent networks of interactions (55). These multivalent interactions can arise from weak interactions between intrinsically disordered regions of the proteins and/or by multivalency through multiple connected domains (25,56). Many stages of RNA metabolism also involve phase separation (56–58), in which RNAs form condensates together with RNA-binding proteins (59). The same cooperativity that enables the formation of phase separated condensates visible under a light microscope will also enable the formation of condensates or aggregates of RNAs and RNA-binding proteins on a nanoscale (60), containing only tens or thousands of molecules, perhaps even containing a single RNA (61,62). Within these aggregates, as well as within true condensates, the concentration of RNA-binding proteins and RNA is much higher than in the cytosol, and therefore even low-affinity binding sites on the RNA can get saturated. We suggest that this type of cooperativity is often amplified by the one we investigate here, involving multiple domains within one protein complex (56). A better quantitative understanding of it could help to give insights into the formation of RNA-protein aggregates and phase-separated condensates.

### Cooperative binding plays a role in other biomolecular interactions

While we focused on RNA binding proteins in this work, the general concept described here is applicable to many other types of interactions. Most closely related might be DNA binding proteins, which employ multi-domain binding in a similar way to RBPs (24,63). The first quantitative treatment of cooperative multivalent binding was applied to antibodies binding to antigens (19). Another application of the presented model could be for binding of proteins to intrinsically disordered regions in proteins (54,64). The same concept of exploiting multivalent binding to maximize avidity is used in fragment based drug discovery (65,66) and in the development of small molecule inhibitors for RNAs (67).

## CONCLUSION

The simple thermodynamic model for RNA binding of multi-domain RBDs shows how cooperative binding of their domains can lead to very high specificity and avidity with RBDs that, alone, have low specificity and affinity. The actual binding motifs of multi-domain RBDs should be considered to be clusters of simple binding motifs, in which the total avidity is determined not only by the affinities of individual motifs but to a large extend by their number and density. A single additional site can change the avidity by two orders of magnitude (Figure 5C), and a twofold change in motif density can change avidity by a factor 10 (Figure 5D).

## DATA AVAILABILITY

The code for the simulations and all calculations is available at https://github.com/soedinglab/cooperative_rbp (permanent DOI: 10.5281/zenodo.7963695). The protein structures used in the validation of the model are available under the PDB accession codes 2n8l, 6dcl, 2adc, 6fq1, 6gqe, 6qey and 2jvz.

## Supplementary Material

lqad057_Supplemental_File
