# Supplementary material for: Cooperativity boosts affinity and specificity of proteins with multiple RNA-binding domains
Source: NAR Genom Bioinform. 2023 Jun 9;5(2):lqad057. doi: 10.1093/nargab/lqad057 (PMC10251633; doi:10.1093/nargab/lqad057)
Supplement: lqad057_Supplemental_File [file lqad057_supplemental_file.pdf]

Supplementary Methods

Cooperativity boosts affinity and specificity of proteins with multiple RNA-binding domains

Simon H. Stitzinger,[1] Salma Sohrabi-Jahromi,[1] and Johannes Söding[1,2,*]

[1]Quantitative and Computational Biology, Max Planck Institute for Biophysical Chemistry, Göttingen, Germany.
[2]Campus-Institut Data Science (CIDAS), Göttingen, Germany.
* Correspondence: soeding@mpinat.mpg.de

1 Calculating avidities for n binding sites

To generalize our approach to n binding sites, we can similarly express the avidity according to the law of mass action in terms of the equilibrium concentrations of all states:

$$K_{\mathrm{av}}(n) := \frac{\sum_{x\neq(0\ldots0)}[x]}{[0\ldots0]\,c} = \sum_{x\neq(0\ldots0)} \frac{[x]}{[0\ldots0]\,c}\,. \tag{1}$$

$[x]$ denotes here the concentration of the x'th state, $[0\ldots0]$ is the concentration of the unbound RNA and c the concentration of the unbound protein. The individual terms can be interpreted as apparent association constants for a theoretical one-step reaction from the unbound state to state x, so that

$$K_{\mathrm{av}}(n) = \sum_{x\neq(0\ldots0)} K_{\mathrm{a},(0\ldots0,x)}\,. \tag{2}$$

The $K_{\mathrm{a},(0\ldots0,x)}$ can be written as a product

$$K_{\mathrm{a},(0\ldots0,x)} = \frac{1}{c}\frac{[x]}{[0\ldots0]} \tag{3}$$

$$= \frac{1}{c}\prod_{i=1}^{|x|}\frac{[p_x(i)]}{[p_x(i-1)]}\,, \tag{4}$$

where $|x|$ is the number of bound sites at state x and p_x describes a path from the unbound state $(0\dots0)$ to state $x = p_x(|x|)$ (for instance flipping unbound sites to bound sites in the order from leftmost to rightmost RNA site). We define $K_{\mathrm{a},p_x(i-1)\rightleftharpoons p_x(i)}$ as the association constant of the reaction between configurations $p_x(i-1)$ and $p_x(i)$ along the path p_x (e.g. Figure 2D, main text). Because for the first factor we can write

$$\frac{[p_x(1)]}{c\,[0\dots0]} = K_{\mathrm{a},(0\dots0)\rightleftharpoons p_x(1)}\,, \tag{5}$$

equation (4) can finally be written as

$$K_{\mathrm{a},(0\dots0,x)} = \prod_{i=1}^{|x|} K_{\mathrm{a},p_x(i-1)\rightleftharpoons p_x(i)}\,. \tag{6}$$

It can readily be seen that

$$K_{\mathrm{av}}(n) = \sum_{x\neq(0\dots0)} \prod_{i=1}^{|x|} K_{\mathrm{a},p_x(i-1)\rightleftharpoons p_x(i)} \tag{7}$$

is a generalization of known results with $n = 2$.

If the individual factors $K_{\mathrm{a},p_x(i-1)\rightleftharpoons p_x(i)} = K_{\mathrm{a},i}\,c_{i-1,i}$ are much larger than 1, which is equivalent to the local concentrations saturating the nearest unbound binding site, the last term of the sum, with the most factors, dominates

$$K_{\mathrm{av}}(n) \approx K_{\mathrm{a},1} \prod_{i=2}^{n} (K_{\mathrm{a},i}\,c_{i-1,i})\,. \tag{8}$$

This shows how each added binding site approximately multiplies the total K_{av} by a factor $K_{\mathrm{a},i}\,c_{i-1,i}$.

2 Effective concentrations using the worm-like chain model

To estimate the effective concentration of a protein domain at an RNA binding site if another domain of the protein already binds the RNA, we model the RNA and the disordered peptide linker between RNA-binding domains as worm-like chains. We use estimates of the persistence length l_p of RNA, which quantifies a chain's stiffness. We call L the chain length between binding sites on the RNA. We call d the distance in 3D space between binding domains on the protein. The local concentration of the second domain at the second RNA binding site can be identified with the probability density of the second RNA site at a 3D distance of d from the first, bound protein domain (Figure 3B, main text). Given a large enough chain length between the binding sites, $L \gg l_\mathrm{p}$, the radial distribution function tends towards a Gaussian distribution,

$$c_{d_i,L_i} = \frac{1}{(2\pi\sigma^2)^{\frac{3}{2}}} \exp\left(-\frac{d_i^2}{2\sigma^2}\right)\,, \tag{9}$$

with a variance of

$$\sigma^2 = \frac{2}{3} l_\mathrm{p} L_i\,. \tag{10}$$

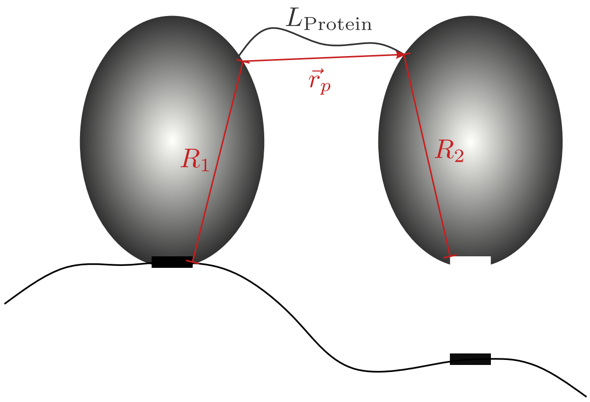

Figure S1: Protein with flexible linker between domains and its RNA target. To describe the effective concentration of the second RNA binding site at the second protein domain, we introduce R_1, R_2 and L_{Protein} as new parameters.

3 Effect of flexible peptide linker on the effective concentration

The above treatment assumes a rigid connection between protein domains. If the protein binding domains are connected by flexible linkers and we allow them to move independently, we need the additional distances R_1, R_2 and L_{Protein} to describe the local concentration, c_{eff} (Figure S1).

It can be expressed by a convolution of three distributions:

$$c_{R_1,R_2,L_{\mathrm{RNA}},L_{\mathrm{Protein}}} \approx \int\int \mathcal{N}\left(\vec{R_1}+\vec{r_p}+\vec{r_2}|0,\sigma^2_{\mathrm{RNA}}\right)\mathcal{N}\left(\vec{r_p}|0,\sigma^2_{\mathrm{Protein}}\right)\frac{\delta(\|\vec{r_2}\|-R_2)}{4\pi R_2^2}\mathrm{d}\vec{r_2}\mathrm{d}\vec{r_p} \tag{11}$$

with

$$\begin{aligned}\sigma^2_{\mathrm{RNA}} &= \frac{2}{3}l_{p,\mathrm{RNA}}\cdot L_{\mathrm{RNA}},\\ \sigma^2_{\mathrm{Protein}} &= \frac{2}{3}l_{p,\mathrm{Protein}}\cdot L_{\mathrm{Protein}}\,.\end{aligned} \tag{12}$$

By integrating equation (11) over $\vec{r_p}$ and defining

$$\sigma^2 = \sigma^2_{\mathrm{RNA}}+\sigma^2_{\mathrm{Protein}}, \tag{13}$$

we can write it as

$$c_{R_1,R_2,L_{\mathrm{RNA}},L_{\mathrm{Protein}}} \approx \int \mathcal{N}\left(\vec{R_1}+\vec{r_2}|0,\sigma^2\right)\frac{\delta(\|\vec{r_2}\|-R_2)}{4\pi R_2^2}\mathrm{d}\vec{r_2} \tag{14}$$

To integrate over the spherical shell with radius R_2, we transform to spherical coordinates

$$c_{R_1,R_2,L_{\mathrm{RNA}},L_{\mathrm{Protein}}} \approx \frac{1}{4\pi R_2^2}\frac{1}{(2\pi\sigma^2)^{\frac{3}{2}}}\int_0^{\pi} \exp\left(-\frac{1}{2\sigma^2}\left(R_1^2+R_2^2-2R_1R_2\cos\theta\right)\right)2\pi R_2^2\sin\theta\mathrm{d}\theta \tag{15}$$

$$= \frac{1}{2(2\pi\sigma^2)^{\frac{3}{2}}}\exp\left(-\frac{1}{2\sigma^2}\left(R_1^2+R_2^2\right)\right)\int_0^{\pi}\exp\left(-\frac{R_1R_2\cos\theta}{\sigma^2}\right)\sin\theta\mathrm{d}\theta \tag{16}$$

$$= \frac{1}{2(2\pi\sigma^2)^{\frac{3}{2}}}\exp\left(-\frac{1}{2\sigma^2}\left(R_1^2+R_2^2\right)\right)\frac{\sigma^2}{R_1R_2}\left(\exp\left(\frac{R_1R_2}{\sigma^2}\right)-\exp\left(-\frac{R_1R_2}{\sigma^2}\right)\right) \tag{17}$$

and finally arrive at

$$c_{R_1,R_2,L_{\mathrm{RNA}},L_{\mathrm{Protein}}} \approx \frac{1}{(2\pi\sigma^2)^{\frac{3}{2}}}\frac{\exp\left(-\frac{1}{2\sigma^2}(R_1-R_2)^2\right)-\exp\left(\frac{1}{2\sigma^2}(R_1+R_2)^2\right)}{2R_1R_2/\sigma^2}\,. \tag{18}$$

4 Procedure for calculating an avidity for "fuzzy" binding and $n = 2$

In analogy to the above described treatment, we write the K_{av} for two domains, when we allow "fuzzy" binding (every domain can bind every other binding site), in terms of the concentrations of all binding configurations as

$$K_{\mathrm{av}}(2) = \frac{[10][01][11][1_20][01_1][1_21_1]}{c\,[00]}\,, \tag{19}$$

where $[1_20]$ and $[01_1]$ denote the concentration of the first domain bound to the second RNA binding site, and the second domain bound to the first binding site, respectively. Again, we can write dissociation constants for individual binding steps. With simple substitutions we finally arrive at

$$K_{\mathrm{a,\,tot}}(2) = K_{\mathrm{a}1}+K_{\mathrm{a}2}+K_{\mathrm{a}1_2}+K_{\mathrm{a}2_1}+c_{d_1,L_1}K_{\mathrm{a}1}K_{\mathrm{a}2}+c_{d_1,L_1}K_{\mathrm{a}1_2}K_{\mathrm{a}2_1}\,, \tag{20}$$

where $K_{\mathrm{a}1_2}$ and $K_{\mathrm{a}2_1}$ denote the association constant for the first domain binding to the second RNA binding site, and the second domain binding to the first RNA binding site, respectively.

5 Simulating cooperative binding with Gillespie stochastic simulation algorithm

The Gillespie algorithm allows us to define model parameters and reactions, and based on these obtain the time dependent concentrations of all species in the system after a

simulation. To build the model we used the Python library Gillespy2. The parameters of the system are as described in the main text.

Before the simulation, all possible molecular entities in the system are defined, which in our case correspond to the different binding configurations. Furthermore, we define all possible reactions between these configurations with rate constants, where the reactions are binding and unbinding events. Because only the ratio between rates determines the end state, the off-rate constant of individual domains k_{off} is set to $k_{\mathrm{off}} = 1$ and according to $K_{\mathrm{d}} = \frac{k_{\mathrm{off}}}{k_{\mathrm{on}}}$ the respective on-rate constant is $k_{\mathrm{on}} = \frac{1}{K_{\mathrm{d}}}$. We assume the rate constants of the unbinding events to be independent of the current binding state. The on-rate constants are calculated based on the current state as $k^*_{\mathrm{on}} = k_{\mathrm{on}} c_{\mathrm{eff}}$ with k_{on} the on-rate constant for the individual domain.

c_{eff} is expressed according to the worm-like-chain model as a Gaussian distribution. When looking at a system with more than 2 binding sites, there are going to be reactions where an unbound binding site is between two bound binding sites as seen in the reaction $101 \rightleftharpoons 111$. The effective concentration in this case has to be expressed according to the laws of probability as the normalized product of two Gaussians. For the normalized product of two Gaussians we get

$$c_{\mathrm{eff}} = \frac{1}{\left(2\pi\sigma_{12}^2\right)^{\frac{3}{2}}} \exp\left(-\frac{(d-\mu_{12})^2}{2\sigma_{12}^2}\right) \tag{21}$$

with

$$\sigma_{12}^2 = \frac{\sigma_1^2\sigma_2^2}{\sigma_1^2+\sigma_2^2} \quad \text{and} \quad \mu_{12} = \frac{\mu_1\sigma_1^2+\mu_2\sigma_2^2}{\sigma_1^2+\sigma_2^2}\,.$$

In our case we have $d = d_1$, $\mu_1 = 0$ and $\mu_2 = d_1 + d_2$ with d_1 and d_2 the distances between binding domains on the protein.

The K_{d} is then calculated based on the appropriate concentrations after reaching equilibrium in the simulation.

6 Model parameters for the comparison of experimental measurements to our theoretical estimates

	K_1/M	K_2/M	RNA/nt	Protein/aa	d/m	R_1/m	R_2/m
ZBP1	2×10^{-6}	1.1×10^{-6}	18	0	3.85×10^{-9}	0	0
hnRNP A1	20.4×10^{-6}	6.8×10^{-6}	4	0	1.99×10^{-9}	0	0
PTB34	2.1×10^{-6}	2×10^{-6}	30	0	2.48×10^{-9}	0	0
IMP3 — RRM12, KH12	9×10^{-6}	4×10^{-6}	6	39	0	5.38×10^{-10}	2.54×10^{-9}
IMP1—KH1,KH2	1.75×10^{-6}	1×10^{-4}	11	0	3.39×10^{-9}	0	0
U2AF	5×10^{-3}	80×10^{-6}	8/4/1	0	0	0	0

7 Calculating K_d values for KSRP

In addition to the examples in the main text of proteins with two domains, for which the K_ds of individual domains and the full-length protein were measured, here, we analyze the four domain protein KSRP, binding to a 34 nt AU-rich RNA [1]. KSRP consists of four KH domains. The second and third domain are linked as a rigid unit, while the first and fourth domain are connected by flexible linkers [2–4]. We predict the avidity of the full length protein and four different variants, in which mutations in individual binding domains remove their ability to bind RNA.

According to the experimental setup, we make the following assumptions in addition to the ones described in the main text.

First, the RNA used in this study does not contain defined binding motifs. Rather, it consists of a stretch of 34 AU-rich nucleotides. The KH-domains do not have a particular sequence specificity for such sequences [3], which is why we assume that they can bind at any position. In line with the assumptions we describe for equation 5 in the main text, we make the approximation that RNA-protein configurations, in which all four domains are bound with minimal sequence length between bound nucleotides on the RNA dominate the population and thus the K_d. This is a result of our model, in which a shorter linker length leads to higher local concentrations, so that the closest possible binding site will saturate at the expense of other binding sites. We only take these binding arrangements into account. We can calculate the number of possible binding arrangements of each protein variant and use equation 5 in the main text to calculate the avidity. The number of possible arrangements is given by

$$(\text{total RNA length} - \text{protein footprint}) \cdot 2\,.$$

The factor 2 is a result of the fact that the domains have no specificity for any motifs in this RNA and the whole protein can thus bind in a 'forward' or 'reverse' orientation. The footprint of the protein is calculated as the sum of all bound nucleotides (4 nt per protein domain) plus the sum of RNA linker lengths L_{ij} between occupied nucleotides. RNA linkers lengths were derived from structural data [2–4]. This results in binding footprints between 14 nt and 22 nt (mutated sequences or full length protein, see tables below).

Second, the study was not able to produce an exact measurement for the K_d of the first domain, which was reported as $> 1\,\mathrm{mM}$. In one case (mutated first domain), this affinity is not needed. In the other three cases, however, we make an educated guess and set $K_{\mathrm{d},1} = 3\,\mathrm{mM}$.

The following tables summarize the parameters we use and the results from the calculations:

Domain	$K_\mathrm{d}/\mu\mathrm{M}$ [5]
KSRP KH1	3000
KSRP KH2	390
KSRP KH3	140
KSRP KH4	350

$L_{\mathrm{RNA},12}$	1 nt
$L_{\mathrm{RNA},23}$	4 nt
$L_{\mathrm{RNA},34}$	1 nt
$L_{\mathrm{Protein},12}$	18 aa
$L_{\mathrm{Protein},34}$	30 aa
d_{23}	3.5×10^{-9} m
R_1	1.6×10^{-9} m
R_{21}	2.3×10^{-9} m
R_{24}	3.1×10^{-9} m
R_{31}	4.0×10^{-9} m
R_{34}	1.8×10^{-9} m
R_4	2.4×10^{-9} m

	Protein footprint/nt	# arrangements	$K_{\mathrm{d,\,exp}}$/nM [1]	$K_{\mathrm{d,\,calc}}$/nM	fold difference
KSRP WT	22	24	3.6	11	3.0
KSRP KH1 Mutant	17	34	7.3	27	3.7
KSRP KH2 Mutant	14	40	43	125	2.9
KSRP KH3 Mutant	14	40	95	225	2.4
KSRP KH4 Mutant	17	34	50	138	2.8

Even though we had to make some assumptions in addition to the main model, the results of this analysis are very self consistent and we are able to replicate all trends that are visible between the measurements of the wildtype and the mutants. These calculations thus further validate our model.

References

(1) Hollingworth, D.; Candel, A. M.; Nicastro, G.; Martin, S. R.; Briata, P.; Gherzi, R.; Ramos, A. KH domains with impaired nucleic acid binding as a tool for functional analysis. *Nucleic Acids Res.* **2012**, *40*, 6873–6886.

(2) Díaz-Moreno, I.; Hollingwort, D.; Kelly, G.; Martin, S.; García-Mayoral, M. F.; Briata, P.; Gherzi, R.; Ramos, A. Orientation of the central domains of KSRP and its implications for the interaction with the RNA targets. *Nucleic Acids Res.* **2010**, *38*, 5193–5205.

(3) García-Mayoral, M. F.; Hollingworth, D.; Masino, L.; Díaz-Moreno, I.; Kelly, G.; Gherzi, R.; Chou, C.-F.; Chen, C.; Ramos, A. The Structure of the C-Terminal KH Domains of KSRP Reveals a Noncanonical Motif Important for mRNA Degradation. *Structure* **2007**, *15*, 485–498.

(4) Díaz-Moreno, I.; Hollingworth, D.; Frenkiel, T. A.; Kelly, G.; Martin, S.; Howell, S.; García-Mayoral, M. F.; Gherzi, R.; Briata, P.; Ramos, A. Phosphorylation-mediated unfolding of a KH domain regulates KSRP localization via 14-3-3 binding. *Nat. Struct. Mol. Biol.* **2009**, *16*, 238–246.

(5) García-Mayoral, M. F.; Díaz-Moreno, I.; Hollingworth, D.; Ramos, A. The sequence selectivity of KSRP explains its flexibility in the recognition of the RNA targets. *Nucleic Acids Res.* **2008**, *36*, 5290–5296.
